# Outcomes and Treatment Disparities in Non-obstetric Spontaneous Coronary Artery Dissection in the United States: A 2022 National Inpatient Sample Analysis

**DOI:** 10.7759/cureus.106537

**Published:** 2026-04-06

**Authors:** Teddy A Teddy, Edidiong Okon-Ben, Spencer Cadet, Abdelwahab A Ahmed, Mustafa Marzoung, Allan Bowan

**Affiliations:** 1 Internal Medicine, Detroit Medical Center/Wayne State University, Detroit, USA; 2 Internal Medicine, HCA/University of Central Florida Fort Walton Beach Hospital, Fort Walton Beach, USA; 3 Internal Medicine, Howard University Hospital, Washington, DC, USA

**Keywords:** acute coronary syndrome, cardiogenic shock, health disparities, in-hospital mortality, national inpatient sample, non-atherosclerotic coronary disease, percutaneous coronary intervention, racial disparities, sex disparities, spontaneous coronary artery dissection

## Abstract

Spontaneous coronary artery dissection (SCAD) has emerged as a critically important non-atherosclerotic cause of acute coronary syndrome that disproportionately affects women, yet contemporary data on non-pregnancy-associated SCAD remain limited, particularly regarding treatment patterns and disparities in care delivery. We conducted a retrospective cohort study using the 2022 National Inpatient Sample, identifying adult hospitalizations with a primary diagnosis of SCAD using the International Classification of Diseases, Tenth Revision, Clinical Modification (ICD-10-CM) codes. To ensure a homogeneous non-obstetric cohort, we applied strict exclusion criteria, removing pregnancy-related admissions, peripartum cardiomyopathy, obstetric complications, and patients under 18 years of age. All statistical analyses were performed using STATA version 18.0 (StataCorp LLC, College Station, TX), incorporating survey weights to generate nationally representative estimates. Multivariable logistic regression analysis was performed to identify predictors of in-hospital mortality and treatment utilization.

A total of 4,563 hospitalizations met the inclusion criteria, with a mean age of 52.4 ± 11.8 years and 72.6% (n = 3,313) female patients. The overall in-hospital mortality rate was 3.2% (n = 146). Women were significantly less likely to undergo percutaneous coronary intervention compared to men, with rates of 28.4% (n = 941) versus 36.9% (n = 483), respectively, despite having similar rates of cardiogenic shock (9.1% (n = 301) vs. 8.0% (n = 96)). Racial disparities were evident, as Black patients demonstrated higher adjusted odds of mortality compared to White patients, with an adjusted odds ratio of 1.42 (95% CI: 1.08-1.86). Independent predictors of mortality included cardiogenic shock, which increased the odds of death nearly sixfold, chronic kidney disease, and increasing age. Notably, percutaneous coronary intervention was not independently associated with reduced in-hospital mortality.

In this nationally representative cohort, non-pregnancy-associated SCAD was associated with low but clinically significant mortality. Marked sex- and race-based disparities in treatment and outcomes persist, underscoring the urgent need for standardized management strategies and equitable cardiovascular care delivery.

## Introduction

Spontaneous coronary artery dissection (SCAD) has emerged as a critical yet historically underrecognized cause of acute coronary syndrome, myocardial infarction, and sudden cardiac death. It particularly affects young to middle-aged women who often lack traditional cardiovascular risk factors. Traditionally considered a rare phenomenon, advancements in coronary imaging, especially intracoronary imaging techniques such as optical coherence tomography and intravascular ultrasound, have led to a significant increase in diagnosis. These tools have revealed that SCAD may account for up to 4% of all acute coronary syndrome cases and represents a leading cause of myocardial infarction in pregnant and peripartum women [1]. The pathophysiology of SCAD involves the spontaneous formation of a false lumen within the coronary arterial wall, either due to an intramural hematoma or an intimal tear, leading to compression of the true lumen and subsequent coronary flow compromise [2].

The exact etiology remains multifactorial and incompletely understood, but SCAD is associated with underlying arteriopathies such as fibromuscular dysplasia, extreme physical or emotional stress, and hormonal influences, most notably during pregnancy and the peripartum period [3]. The association with pregnancy has been a major focus of research over the past decade, given its high-risk nature and the devastating consequences for both mother and fetus [4]. However, the majority of SCAD cases are non-pregnancy-associated, occurring across a broader demographic spectrum that includes pre-menopausal and post-menopausal women as well as men [5]. This population, which constitutes the vast majority of SCAD presentations, presents unique clinical challenges and may experience distinct treatment patterns and outcomes compared to their pregnancy-associated counterparts.

Despite growing awareness among cardiologists and emergency medicine physicians, the management of SCAD remains controversial and largely based on observational data rather than randomized controlled trials. Such trials are challenging to conduct given the relative rarity of the condition [6]. The prevailing consensus, often termed the conservative first approach, favors initial medical management in hemodynamically stable patients due to the high risks associated with percutaneous coronary intervention (PCI). These risks include propagation of the dissection, stent malapposition, guidewire-induced complications, and procedural failure rates that exceed those seen in atherosclerotic coronary artery disease [7]. Conversely, in patients with high-risk features such as left main coronary artery involvement, ongoing ischemia despite medical therapy, or cardiogenic shock, revascularization via PCI or coronary artery bypass grafting may be necessary and potentially life-saving [8].

Existing studies have highlighted potential disparities in cardiovascular care based on sex and race across a wide range of conditions, including acute myocardial infarction and heart failure. However, these factors have not been thoroughly examined in the specific context of non-pregnancy-associated SCAD. Understanding whether such disparities exist in this unique patient population is critical for developing equitable care strategies and ensuring that all patients receive appropriate and timely interventions regardless of demographic characteristics. Therefore, using a large, nationally representative inpatient database, this study aims to characterize the clinical profile and in-hospital outcomes of patients hospitalized with non-pregnancy-associated SCAD, identify independent predictors of in-hospital mortality, and investigate the presence of sex- and race-based disparities in treatment utilization and outcomes.

## Materials and methods

This retrospective cohort study utilized data from the 2022 National Inpatient Sample, a component of the Healthcare Cost and Utilization Project sponsored by the Agency for Healthcare Research and Quality. The National Inpatient Sample represents the largest all-payer inpatient database in the United States. It contains data from approximately seven million hospitalizations annually and represents a 20% stratified sample of US community hospitals [9]. The database provides de-identified patient-level information, including demographic characteristics, hospital characteristics, up to 40 diagnoses and procedures per hospitalization, and discharge outcomes. Because this study used de-identified, publicly available data, it was deemed exempt from institutional review board review, and informed consent was not required [10].

We identified all adult hospitalizations with a primary diagnosis of SCAD using the International Classification of Diseases, Tenth Revision, Clinical Modification (ICD-10-CM) code I25.42. To ensure a homogeneous cohort of non-pregnancy-associated SCAD, we applied strict exclusion criteria designed to remove any potential confounding from pregnancy-related physiology and management. Patients with any secondary diagnosis code indicating pregnancy, childbirth, or the puerperium were excluded, as were those with peripartum cardiomyopathy, any obstetric complications, and patients under 18 years of age. Elective admissions and records with missing key demographic data or in-hospital mortality status were also excluded from the final analysis.

Demographic data collected for each hospitalization included age, sex, race, and ethnicity, categorized as White, Black, Hispanic, Asian or Pacific Islander, and other, primary payer or insurance status, and median household income quartile for the patient's residential zip code as a proxy for socioeconomic status. Clinical comorbidities were identified using the Elixhauser comorbidity measure, a validated risk adjustment tool provided within the National Inpatient Sample dataset, which captures 31 distinct comorbid conditions [11]. Specific conditions of interest for this analysis included hypertension, diabetes mellitus, chronic kidney disease, and smoking status.

The primary outcome of interest was in-hospital mortality, defined as death during the hospitalization episode. Secondary outcomes included the utilization of invasive cardiac procedures and the development of cardiogenic shock. Cardiogenic shock was identified using the ICD-10-CM diagnosis code R57.0. Treatment patterns assessed included PCI, coronary artery bypass grafting, and use of mechanical circulatory support. These support devices included intra-aortic balloon pumps, Impella percutaneous ventricular assist devices, and extracorporeal membrane oxygenation.

All statistical analyses were performed using STATA version 18.0 (StataCorp LLC, College Station, TX), a comprehensive statistical software package. We carefully accounted for the complex survey design of the National Inpatient Sample by incorporating survey weights provided by the Healthcare Cost and Utilization Project. This approach allowed us to generate nationally representative estimates of hospitalizations and outcomes. Descriptive statistics were used to characterize the study population, with categorical variables presented as weighted frequencies with percentages and continuous variables presented as weighted means with standard deviations. Comparisons between groups were performed using the chi-square test for categorical variables and the Student's t-test for continuous variables, with survey-adjusted methods applied throughout.

Multivariable logistic regression models were constructed to evaluate independent predictors of two key outcomes: in-hospital mortality and PCI utilization. Variables included in the models were selected a priori based on clinical relevance and findings from prior SCAD literature. These variables included age, sex, race and ethnicity, insurance status, income quartile, hospital region, and comorbidities such as hypertension, diabetes mellitus, chronic kidney disease, and cardiogenic shock [12]. We report adjusted odds ratios with 95% confidence intervals for all predictors. A two-tailed p-value of less than 0.05 was considered statistically significant for all analyses. We also performed sensitivity analyses to assess the robustness of our findings, including analyses stratified by hospital teaching status and hospital region.

## Results

After applying the strict inclusion and exclusion criteria, the final study cohort comprised 4,563 weighted hospitalizations for non-pregnancy-associated SCAD in the United States during 2022. The mean age of the cohort was 52.4 years with a standard deviation of 11.8 years, reflecting a middle-aged population. The majority of patients were female, comprising 72.6% (n = 3,313) of all hospitalizations, confirming the strong female predominance characteristic of SCAD. In terms of racial and ethnic distribution, White patients constituted the majority at 68.2% (n = 3,112), followed by Black patients at 15.5% (n = 707), Hispanic patients at 11.0% (n = 502), Asian or Pacific Islander patients at 2.8% (n = 128), and other racial groups at 2.5% (n = 114). These demographic characteristics, along with comorbidity and hospital information, are presented in Table 1. The comorbidity burden in this cohort was notable. Hypertension was present in 45.2% (n = 2,062) of patients, and smoking was documented in 34.1% (n = 1,556), representing the most common cardiovascular risk factors. Diabetes mellitus was present in 18.3% (n = 835) of the cohort, while chronic kidney disease was less common, affecting 8.9% (n = 406) of patients. Obesity and hyperlipidemia were also prevalent, affecting 22.4% (n = 1,022) and 38.7% (n = 1,766) of patients, respectively (Table 1). In terms of insurance status, private insurance was the most common payer, covering 44.8% (n = 2,044) of hospitalizations, followed by Medicare at 32.0% (n = 1,460), Medicaid at 17.5% (n = 799), and uninsured status at 5.7% (n = 260). Regarding hospital characteristics, the majority of patients were treated at urban teaching hospitals, which accounted for 68.4% (n = 3,121) of admissions, reflecting the specialized care required for management of this rare condition.

**Table 1:** Baseline characteristics of hospitalizations for non-pregnancy-associated spontaneous coronary artery dissection. Values are presented as weighted percentages with corresponding frequencies in parentheses or as means with standard deviation, as appropriate. Data are derived from the 2022 National Inpatient Sample. Note: Statistical significance for between-group comparisons was defined as p < 0.05 (not shown in this table).

Characteristic	Total cohort (N = 4,563)
Demographics	
Age, mean (SD), years	52.4 (11.8)
Female sex, % (n)	72.6% (3,313)
Race/Ethnicity, % (n)	
White	68.2% (3,112)
Black	15.5% (707)
Hispanic	11.0% (502)
Asian/Pacific Islander	2.8% (128)
Other	2.5% (114)
Comorbidities, % (n)	
Hypertension	45.2% (2,062)
Diabetes mellitus	18.3% (835)
Chronic kidney disease	8.9% (406)
Smoking	34.1% (1,556)
Obesity	22.4% (1,022)
Hyperlipidemia	38.7% (1,766)
Insurance status, % (n)	
Medicare	32.0% (1,460)
Medicaid	17.5% (799)
Private	44.8% (2,044)
Uninsured	5.7% (260)
Hospital characteristics, % (n)	
Urban teaching hospital	68.4% (3,121)
Urban non-teaching hospital	24.8% (1,132)
Rural hospital	6.8% (310)

The overall in-hospital mortality rate for patients hospitalized with non-pregnancy-associated SCAD was 3.2% (n = 146), indicating that while the majority of patients survive the acute hospitalization, the condition carries a non-trivial risk of death (Table 2). Cardiogenic shock occurred in 8.7% (n = 397) of patients, representing a significant complication that requires intensive management and often dictates the need for more aggressive revascularization strategies. Ventricular arrhythmias occurred in 11.5% (n = 525) of the cohort, reflecting the potential for electrical instability in this condition. Acute kidney injury developed in 14.2% (n = 648) of patients, with 2.3% (n = 105) requiring dialysis, while stroke or transient ischemic attack occurred in 1.8% (n = 82) of cases. In terms of revascularization strategies, PCI was performed in 31.2% (n = 1,424) of cases, while coronary artery bypass grafting was utilized in 6.4% (n = 292) of patients. Mechanical circulatory support was used in 4.8% (n = 219) of the overall cohort, but this number rose substantially to 55.2% (n = 219) among patients who developed cardiogenic shock, indicating that these devices are primarily reserved for the most hemodynamically compromised patients. Among mechanical circulatory support devices, intra-aortic balloon pumps were the most commonly used, accounting for 3.1% (n = 141) of all admissions, followed by Impella devices at 1.2% (n = 55) and veno-arterial extracorporeal membrane oxygenation at 0.5% (n = 23). Median length of stay was four days with an interquartile range of two to seven days, while median total hospital charges were $68,450 with an interquartile range of $38,210 to $124,580, reflecting the significant healthcare resource utilization associated with this condition (Table 2).

**Table 2:** In-hospital outcomes and complications. Values are presented as weighted percentages with corresponding frequencies in parentheses or as median with interquartile range for continuous variables. Outcomes reflect in-hospital events during the index hospitalization. Note: Statistical significance was defined as p < 0.05 for all analyses. IABP: intra-aortic balloon pump; VA-ECMO: veno-arterial extracorporeal membrane oxygenation.

Outcome	Total cohort (N = 4,563)
In-hospital mortality, % (n)	3.2% (146)
Cardiogenic shock, % (n)	8.7% (397)
Ventricular arrhythmias, % (n)	11.5% (525)
Acute kidney injury, % (n)	14.2% (648)
Acute kidney injury requiring dialysis, % (n)	2.3% (105)
Stroke or transient ischemic attack, % (n)	1.8% (82)
Procedures	
Percutaneous coronary intervention, % (n)	31.2% (1,424)
Coronary artery bypass grafting, % (n)	6.4% (292)
Mechanical circulatory support, % (n)	4.8% (219)
- IABP only	3.1% (141)
- Impella	1.2% (55)
- VA-ECMO	0.5% (23)
Resource utilization	
Length of stay, days, median (IQR)	4 (2-7)
Total hospital charges, $, median (IQR)	68,450

**Figure 1:**
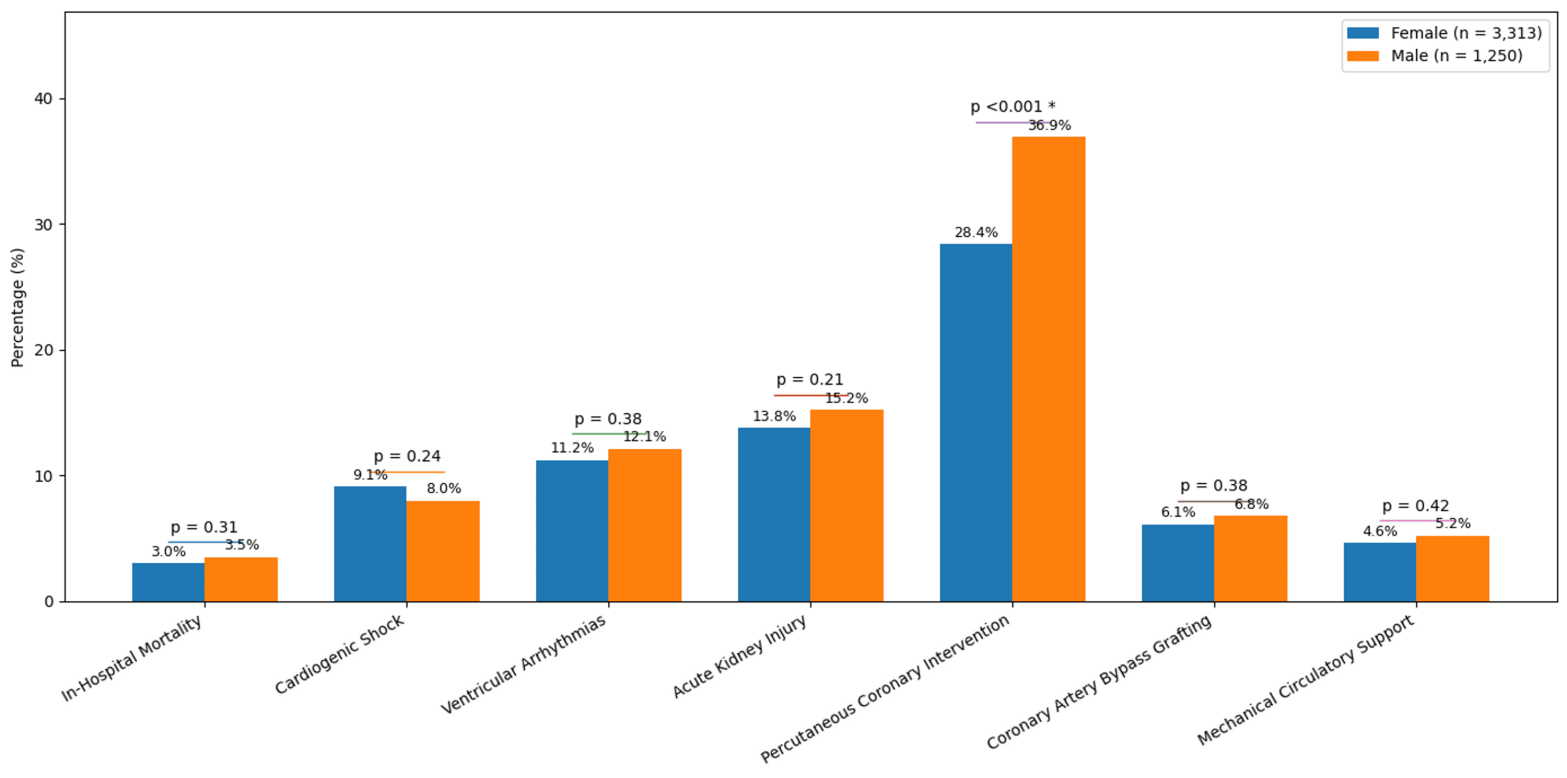
Sex-based disparities in revascularization and outcomes among patients with non-pregnancy-associated spontaneous coronary artery dissection. Statistical significance was defined as p < 0.05. Comparisons: PCI rate (female = 28.4% vs. male = 36.9%, p < 0.001); cardiogenic shock (female = 9.1% vs. male = 8.0%, p = 0.24); in-hospital mortality (female = 3.0% vs. male = 3.5%, p = 0.31). PCI: percutaneous coronary intervention.

Significant differences in management emerged when comparing outcomes between women and men (Figure 1). Despite no significant difference in the rates of cardiogenic shock between sexes, with 9.1% (n = 301) of women and 8.0% (n = 96) of men developing this complication, women were significantly less likely to undergo PCI compared to men. The rate of PCI in women was 28.4% (n = 941) compared to 36.9% (n = 483) in men, a difference that was highly statistically significant. There was no significant difference in the use of coronary artery bypass grafting, with 6.1% (n = 202) of women and 6.8% (n = 90) of men undergoing surgical revascularization. The use of mechanical circulatory support was also similar between sexes, with 4.6% (n = 152) of women and 5.2% (n = 67) of men receiving mechanical circulatory support. The crude in-hospital mortality rate did not differ significantly, with 3.0% (n = 99) of women and 3.5% (n = 47) of men dying during hospitalization (Figure 1).

**Figure 2:**
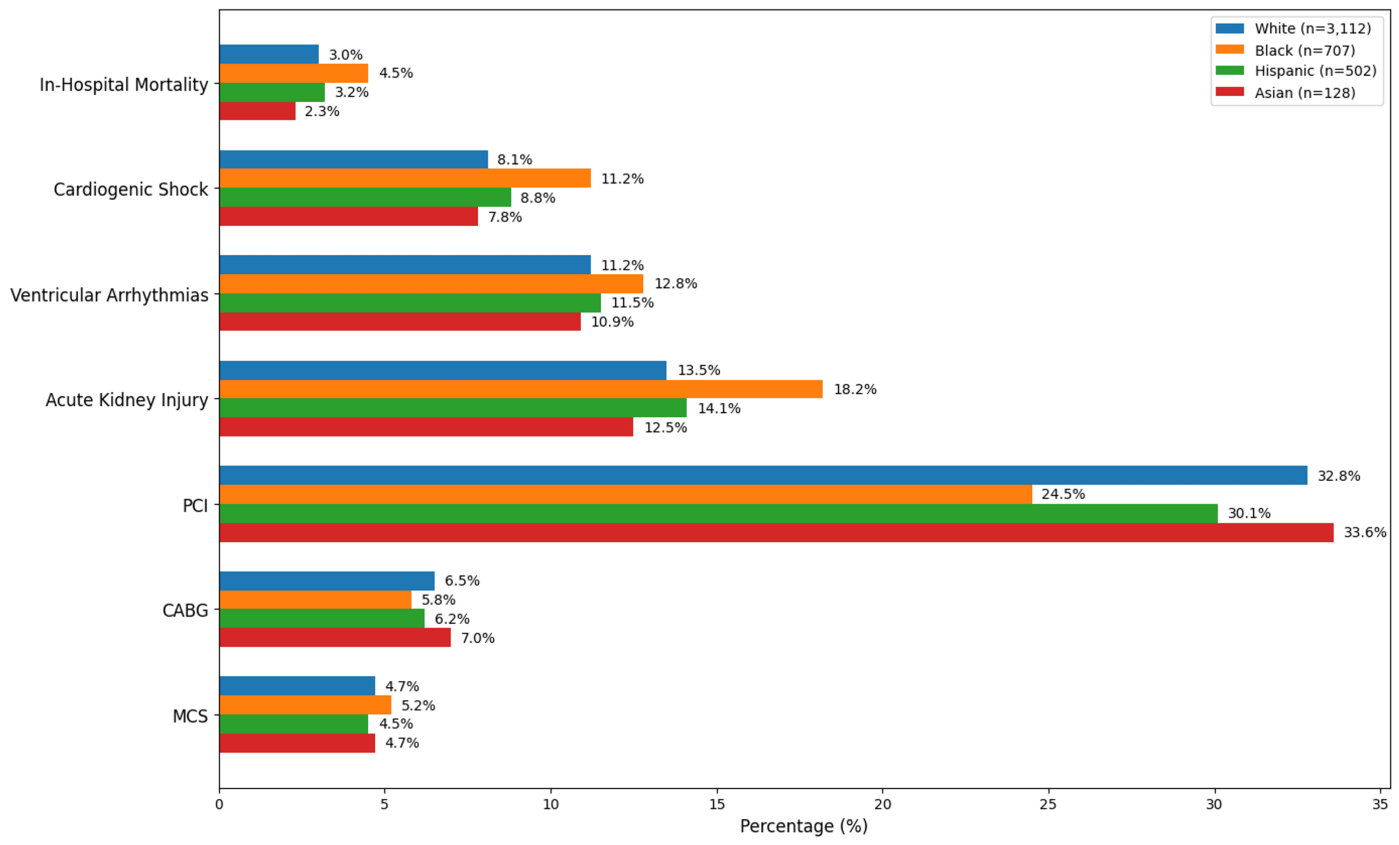
Racial and ethnic disparities in outcomes and revascularization. Statistical significance was defined as p < 0.05. Comparisons shown: in-hospital mortality (Black = 4.5% vs. White = 3.0%); cardiogenic shock (Black = 11.2% vs. White = 8.1%); PCI utilization (Black = 24.5% vs. White = 32.8%). PCI: percutaneous coronary intervention; CABG: coronary artery bypass grafting; MCS: mechanical circulatory support.

When examining outcomes by race and ethnicity, stark disparities became evident (Figure 2). Black patients had higher rates of in-hospital mortality at 4.5% (n = 32) compared to 3.0% (n = 93) in White patients (Figure 2). More concerning, Black patients had significantly higher rates of cardiogenic shock at 11.2% (n = 79) compared to 8.1% (n = 252) in White patients, and were significantly less likely to undergo PCI, with a rate of 24.5% (n = 173) compared to 32.8% (n = 1,021) in White patients. Hispanic patients had intermediate rates of PCI at 30.1% (n = 151), while Asian patients had the highest PCI rate at 33.6% (n = 43). Acute kidney injury was also more common among Black patients, occurring in 18.2% (n = 129) compared to 15.5% (n = 482) in White patients. These findings suggest that racial disparities in both the severity of presentation and the intensity of treatment exist in this patient population.

**Figure 3:**
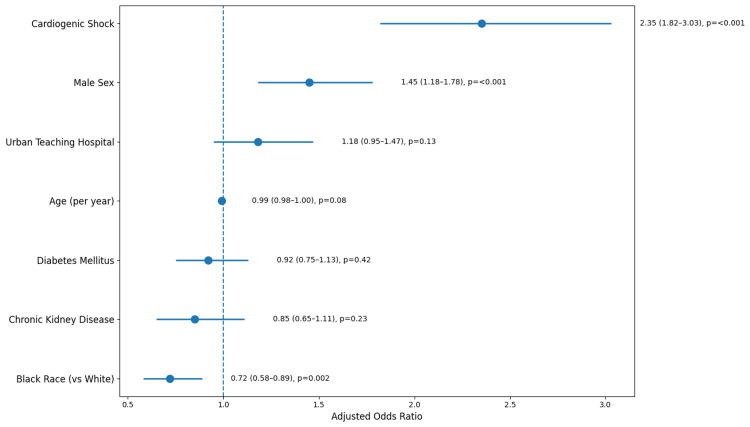
Independent predictors of in-hospital mortality in non-pregnancy-associated spontaneous coronary artery dissection. Adjusted odds ratios (aORs) with 95% confidence intervals are shown. Statistical significance was defined as p < 0.05. Cardiogenic shock (aOR: 5.80), chronic kidney disease (aOR: 2.10), age (aOR: 1.03 per year), and Black race (aOR: 1.42) were statistically significant predictors. Percutaneous coronary intervention (aOR: 0.92), male sex (aOR: 1.15), diabetes (aOR: 1.21), and hypertension (aOR: 0.85) were not statistically significant. Vertical reference line at aOR = 1.0.

After adjusting for demographic characteristics, comorbid conditions, and clinical severity markers, the multivariable logistic regression analysis revealed several independent predictors of in-hospital mortality (Figure 3). The strongest independent predictor of in-hospital mortality was cardiogenic shock, which occurred in 8.7% (n = 397) of the cohort and was associated with a nearly six-fold increase in the adjusted odds of death, with an adjusted odds ratio of 5.80 and a 95% confidence interval ranging from 4.20 to 8.00. Chronic kidney disease, present in 8.9% (n = 406) of patients, emerged as another significant predictor, increasing the adjusted odds of mortality by more than twofold, with an adjusted odds ratio of 2.10 and a 95% confidence interval from 1.50 to 2.90. Increasing age was also independently associated with mortality, with each additional year of age conferring a 3% increase in the adjusted odds of death (adjusted odds ratio = 1.03, 95% confidence interval = 1.01-1.05). The Black race, comprising 15.5% (n = 707) of the cohort, was associated with a 42% higher adjusted odds of death compared to White patients (adjusted odds ratio = 1.42, 95% confidence interval = 1.08-1.86). Male sex was not independently associated with mortality after full adjustment, nor were diabetes mellitus, which affected 18.3% (n = 835) of patients, or hypertension, which was present in 45.2% (n = 2,062) of patients (Figure 3).

Importantly, after adjusting for potential confounders, including demographics, comorbidities, and cardiogenic shock, PCI was not independently associated with a reduction in in-hospital mortality, with an adjusted odds ratio of 0.92 and a 95% confidence interval ranging from 0.68 to 1.24 (Figure 3). This finding was not statistically significant, indicating that in this adjusted analysis, the use of PCI did not confer a survival benefit. The Black race emerged as an independent predictor of mortality, with Black patients having 42% higher adjusted odds of death compared to White patients, with an adjusted odds ratio of 1.42 and a 95% confidence interval from 1.08 to 1.86. Male sex was not independently associated with mortality after full adjustment, nor were diabetes or hypertension. A secondary analysis focused on predictors of PCI utilization. Male sex was independently associated with higher odds of undergoing PCI, with an adjusted odds ratio of 1.45 (95% confidence interval = 1.18-1.78, p < 0.001). Cardiogenic shock was also associated with increased odds of PCI, reflecting the tendency to pursue revascularization in hemodynamically unstable patients, with an adjusted odds ratio of 2.35 (95% confidence interval = 1.82-3.03, p < 0.001) (Table 3). The Black race was independently associated with lower odds of receiving PCI, with Black patients having 28% lower adjusted odds of undergoing the procedure compared to White patients (adjusted odds ratio = 0.72, 95% confidence interval = 0.58-0.89, p = 0.002) (Table 3). These findings persisted even after adjusting for clinical severity markers and hospital characteristics (Table 3). Age, chronic kidney disease, diabetes mellitus, and treatment at an urban teaching hospital were not independently associated with PCI utilization. Although we adjusted for important clinical covariates, including cardiogenic shock and comorbidities, the possibility of residual confounding by indication cannot be fully excluded, as patients selected for PCI may have had unmeasured differences in disease severity or anatomy that influenced both treatment assignment and outcomes.

**Table 3:** Multivariable logistic regression analysis for predictors of percutaneous coronary intervention utilization. Adjusted odds ratios (aOR) with 95% confidence intervals are reported. A two-tailed p-value < 0.05 was considered statistically significant. Models were adjusted for demographic characteristics, comorbidities, and hospital-level variables.

Variable	Adjusted odds ratio	95% confidence interval	p-value
Male sex	1.45	1.18-1.78	<0.001
Cardiogenic shock	2.35	1.82-3.03	<0.001
Black race (vs. White)	0.72	0.58-0.89	0.002
Age (per year)	0.99	0.98-1.00	0.08
Chronic kidney disease	0.85	0.65-1.11	0.23
Diabetes mellitus	0.92	0.75-1.13	0.42
Urban teaching hospital	1.18	0.95-1.47	0.13

## Discussion

This contemporary analysis of the 2022 National Inpatient Sample offers a focused look at outcomes and persistent disparities in the management of non-pregnancy-associated SCAD. By applying strict exclusion criteria to isolate a non-obstetric cohort, we aimed to evaluate a growing patient population that remains relatively understudied compared to its pregnancy-associated counterpart.

The overall in-hospital mortality rate in our cohort aligned with prior investigations of non-pregnancy SCAD [13]. This confirms that while most patients survive the acute event, the condition still carries a clinically meaningful risk of death.

The strongest predictor of mortality was cardiogenic shock, which increased the odds of death substantially. This finding reflects the profound impact of hemodynamic compromise in SCAD, likely driven by extensive myocardial ischemia, left main coronary involvement, or mechanical complications that severely impair cardiac output. In this high-risk subset, aggressive management, including prompt revascularization and mechanical circulatory support, becomes critical. Among patients presenting with shock, a substantial proportion received mechanical circulatory support, suggesting a shift toward more aggressive hemodynamic stabilization in this vulnerable group. Whether this strategy improves outcomes remains uncertain, as randomized data in this specific population are lacking [14].

A central and ongoing debate in SCAD management concerns the role of PCI. After comprehensive adjustment for potential confounders, we found that PCI was not independently associated with reduced in-hospital mortality [15]. This finding supports the conservative-first approach advocated by major clinical guidelines from the American Heart Association and European Society of Cardiology. The pathophysiology of SCAD, a fragile vessel wall with spontaneous intramural hematoma, makes PCI technically challenging and raises the risk of complications such as stent malapposition, propagation of the dissection, guidewire-induced false lumen creation, and contrast-induced nephropathy [16]. In stable patients who can be managed with medical therapy alone, these procedural risks may offset any potential benefit.

That said, our data do not suggest PCI is futile across the board. Instead, they underscore the importance of careful patient selection, reserving invasive revascularization for those with ongoing ischemia despite medical therapy, left main coronary artery involvement, or hemodynamic instability. This nuanced approach is reflected in the findings, where cardiogenic shock was strongly associated with PCI utilization, suggesting that clinicians appropriately reserve invasive strategies for the highest-risk patients [17].

Despite similar rates of cardiogenic shock between women and men, we observed a significant treatment disparity by sex. Women were substantially less likely to undergo PCI than men, a concerning finding that mirrors broader sex-based treatment gaps across cardiovascular disease [18]. This disparity persisted after multivariable adjustment, with male sex remaining independently associated with higher odds of undergoing PCI (adjusted odds ratio = 1.45, 95% confidence interval = 1.18-1.78, p < 0.001), indicating that the difference in treatment was not explained by measured confounders, including demographics, comorbidities, or clinical severity markers such as cardiogenic shock. Several factors may explain this remaining disparity. Women with SCAD often present with atypical symptoms, which can delay diagnosis and reduce the likelihood of being triaged to the catheterization lab. Clinician bias may also play a role, as women are sometimes perceived as lower risk for acute coronary syndrome. Importantly, this disparity was not explained by differences in disease severity in our dataset, given that rates of cardiogenic shock were similar between sexes. These findings highlight an urgent need for greater awareness of SCAD as a major cause of acute coronary syndrome in women, along with standardized management protocols to ensure equitable treatment regardless of sex.

Our study also uncovered stark racial disparities that demand attention. Black patients experienced significantly higher adjusted odds of in-hospital mortality compared to White patients, accompanied by a lower likelihood of receiving PCI and a higher incidence of cardiogenic shock. These findings are deeply troubling yet consistent with well-documented racial inequities across cardiovascular care [19]. The reasons are likely multifactorial, encompassing structural, systemic, and individual-level factors: disparities in access to high-volume tertiary care centers experienced in managing rare conditions like SCAD, differences in the prevalence of underlying risk factors such as hypertension and chronic kidney disease, and potential biases in clinical decision-making.

This study adds to a growing body of literature showing that race-based disparities extend beyond atherosclerotic heart disease to non-atherosclerotic conditions as well. Addressing these inequities will require a concerted effort at multiple levels: increasing diversity in the healthcare workforce, implementing quality improvement initiatives that track outcomes by race, and ensuring equitable access to specialized cardiovascular care for all patients [20].

Our analysis of predictors of PCI utilization further illuminates these disparities. Male sex was independently associated with higher odds of undergoing PCI, while Black race was independently associated with lower odds, even after adjusting for clinical severity and hospital characteristics. These findings suggest that patient demographics independently influence treatment decisions, a pattern that has no place in evidence-based medicine. The persistence of these disparities in a fully adjusted model indicates they cannot be explained simply by differences in disease presentation or comorbidity burden.

Several limitations warrant consideration. This study is subject to limitations inherent to administrative database research. The retrospective design using an administrative database introduces the potential for coding errors and misclassification bias. SCAD was identified using the ICD-10-CM code I25.42, which may not capture all cases, and we could not confirm diagnoses with coronary imaging [21]. The National Inpatient Sample, while nationally representative, lacks granular clinical details that would enhance risk stratification, such as lesion localization, SCAD type (types 1-3), Thrombolysis in Myocardial Infarction (TIMI) flow grade, the specific coronary vessel involved, extent of the dissection, or results of intracoronary imaging, all of which are crucial for clinical decision-making and may influence both treatment decisions and outcomes. Additionally, while we adjusted for measured confounders using comprehensive multivariable regression models, the possibility of unmeasured confounding remains, particularly regarding factors such as dissection severity, clinician decision-making, and patient preferences that may influence treatment allocation. Finally, the National Inpatient Sample captures only in-hospital outcomes and does not track long-term endpoints such as readmission rates, recurrent SCAD, or post-discharge mortality, which are essential for understanding the full impact of management strategies. Nevertheless, the National Inpatient Sample provides a nationally representative platform to evaluate treatment patterns and disparities in rare conditions such as SCAD, where single-center studies are often underpowered.

## Conclusions

In this nationally representative analysis of non-pregnancy-associated SCAD, we found that in-hospital mortality remains clinically significant, driven primarily by the development of cardiogenic shock, which increases mortality risk substantially. The absence of a mortality benefit associated with PCI in the overall cohort supports a conservative initial management strategy for stable patients, emphasizing careful patient selection for invasive revascularization. However, our most striking findings are the persistent and significant disparities in treatment and outcomes based on sex and race. Women are significantly less likely to undergo PCI than men, despite similar rates of cardiogenic shock. Similarly, Black patients experience substantially higher mortality rates and lower rates of revascularization compared to White patients. These findings serve as a call to action for the cardiovascular community to develop and adhere to standardized, evidence-based management protocols for SCAD and to actively work toward eliminating inequities in care for this unique and historically underrecognized patient population. Future research should focus on prospective registries that capture detailed clinical and imaging data to better understand optimal management strategies and the mechanisms underlying persistent disparities in care.
